# An analysis of near misses identified by anesthesia providers in the intensive care unit

**DOI:** 10.1186/s12871-015-0075-z

**Published:** 2015-06-17

**Authors:** Angela K.M. Lipshutz, James E. Caldwell, David L. Robinowitz, Michael A. Gropper

**Affiliations:** Department of Anesthesia and Perioperative Care, University of California, San Francisco, 505 Parnassus Avenue, San Francisco, CA 94143 USA

**Keywords:** Incident reporting, Adverse event, Near miss, Critical care, Critical care anesthesiology, Airway management

## Abstract

**Background:**

Learning from adverse events and near misses may reduce the incidence of preventable errors. Current literature on adverse events and near misses in the ICU focuses on errors reported by nurses and intensivists. ICU near misses identified by anesthesia providers may reveal critical events, causal mechanisms and system weaknesses not identified by other providers, and may differ in character and causality from near misses in other anesthesia locations.

**Methods:**

We analyzed events reported to our anesthesia near miss reporting system from 2009 to 2011. We compared causative mechanisms of ICU near misses with near misses in other anesthesia locations.

**Results:**

A total of 1,811 near misses were reported, of which 22 (1.2 %) originated in the ICU. Five causal mechanisms explained over half of ICU near misses. Compared to near misses from other locations, near misses from the ICU were more likely to occur while on call (45 % vs. 19 %, *p* = 0.001), and were more likely to be associated with airway management (50 % vs. 12 %, *p* < 0.001). ICU near misses were less likely to be associated with equipment issues (23 % vs. 48 %, *p* = 0.02).

**Conclusions:**

A limited number of causal mechanisms explained the majority of ICU near misses, providing targets for quality improvement. Errors associated with airway management in the ICU may be underappreciated. Specialist consultants can identify systems weaknesses not identified by critical care providers, and should be engaged in the ICU patient safety movement.

## Background

Preventable errors are estimated to cause nearly 100,000 deaths in the United States each year, and cost the healthcare system $9 billion annually [1]. Errors occur more frequently in the intensive care unit (ICU) than other areas of the hospital due to the acuity of illness and the frequency and complexity of interventions [2]. Incident reporting systems and protocols for the analysis of patient safety events are essential components of ICU patient safety programs, [3] since learning from adverse events may reduce the incidence of preventable errors.

Current literature on adverse events and near misses in the ICU primarily relies on events reported by critical care nurses and intensivist physicians [4–9]. Anesthesia providers also play an important role in caring for the critically ill, not only as members of the ICU team, but in the provision of anesthesia for procedures performed in the ICU, transport and handoff of patients traveling to or from procedures outside the ICU, and response to emergencies requiring airway management or cardiopulmonary resuscitation. To our knowledge, no study has focused solely on patient safety events in the ICU from the unique perspective of the anesthesiologist.

We hypothesized that analysis of patient safety events in the ICU identified by anesthesia providers may reveal critical events, causal mechanisms, and system weaknesses not identified in previous literature on the topic. Furthermore, patient safety events in the ICU may differ in character and causal mechanisms from events identified by anesthesia providers in other locations in the hospital, underscoring the important and unique role of the anesthesiologist in care of the critically ill. Thus, we sought to characterize near miss reports submitted by anesthesia providers in the ICU to our department’s near miss reporting system and compare them to reports submitted by anesthesia providers in other hospital locations in order to highlight unidentified weaknesses in the provision of critical care and provide targets for quality improvement.

## Methods

### Setting

The University of California, San Francisco (UCSF) Medical Center is a 560-bed academic quaternary care hospital with 77 adult ICU beds (32 medical/surgical, 29 neuroscience, and 16 cardiology/cardiothoracic surgery) and 76 pediatric ICU beds (17 medical/surgical, 8 cardiology/cardiothoracic surgical, 51 neonatal). The adult medical/surgical ICUs are closed for malignant hematology patients and patients of surgical subspecialists, such that the ICU team takes primary care of approximately one-third of ICU admissions. For all other patients, the adult medical/surgical ICU is semi-closed—primary care of the patient is the responsibility of the admitting service, but intensivist consultation is mandatory. In the adult neuroscience and cardiology/cardiothoracic ICUs, there is mandatory intensivist consultation only for patients requiring mechanical ventilation; intensivist consultation for other patients occurs at the discretion of the primary team. The typical adult critical care team includes residents from the departments of medicine, anesthesia, and emergency medicine; a critical care fellow from the department of medicine, surgery, anesthesia, emergency medicine, or neurology; and a board-certified or board-eligible intensivist from the department of medicine, surgery, or anesthesia. Intensivist coverage is provided by anesthesia intensivists 60 % of the time. All of the pediatric and neonatal ICUs are closed, with care provided by critical care trained pediatric intensivists in conjunction with pediatric critical care fellows, neonatology fellows, and pediatric residents.

The UCSF Department of Anesthesia and Perioperative Care provides anesthesia services at six sites in San Francisco: a 560-bed tertiary care academic medical center (UCSF Medical Center), a 564-bed hospital and trauma center owned and operated by the city and county of San Francisco, a 90-bed academic hospital and comprehensive cancer center, a 104-bed Veteran’s Affairs Hospital, a six-operating room outpatient surgery center associated with UCSF Medical Center, and a four-operating room stand alone orthopedic surgery center. The department is housed at the UCSF Medical Center and the majority of clinical care occurs there, in the operating rooms, preoperative and postoperative areas, labor and delivery, remote locations, and the ICU. The department includes 24 board-certified intensivists, who attend in the adult medical/surgical ICUs as well as the adult subspecialty ICUs at UCSF Medical Center. Anesthesia residents and anesthesia critical care fellows rotate through the adult ICUs during their training. Anesthesia providers do not staff the pediatric ICUs. In addition to the role of anesthesia providers on the adult critical care team described above, anesthesia providers may also be involved in adult or pediatric ICU patient care during patient handoffs (transport to or from procedures), provision of anesthesia for procedures performed in the ICU, airway emergencies, and code blues.

### Data source

**Fig. 1:**
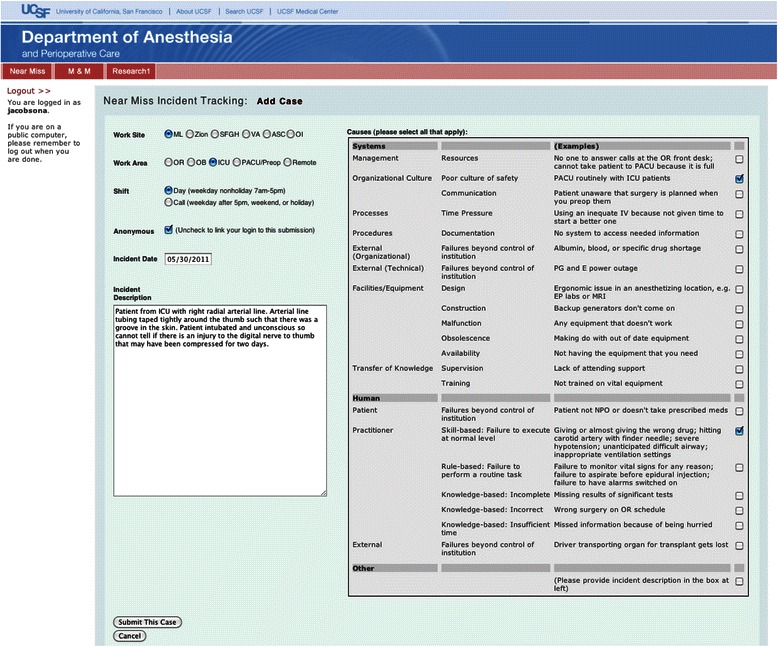
Near miss reporting system user interface. ML, Moffitt Long Hospital; Zion, Mount Zion Hospital; SFGH, San Francisco General Hospital; VA, San Francisco Veteran’s Affairs Hospital; ASC, Ambulatory Surgery Center; OI, Orthopedic Institute; OR, operating room; OB, obstetrical ward; ICU, intensive care unit; PACU/Preop, Post-operative and pre-operative care units; IV, intravenous line; PG and E, Pacific Gas and Electric; EP, electrophysiology; MRI, magnetic resonance imaging; NPO, nil per os (nothing by mouth)

On January 1, 2009, the department instituted a near miss reporting system in order to capture and study potential incidents that occur during the provision of anesthesia. Near miss reports are submitted by anesthesia providers (faculty, certified registered nurse anesthetists, residents, and fellows) via a voluntary, anonymous, self-reporting, internet-based near miss reporting system (Fig. 1). Near miss reports can be submitted by providers at any of the six sites. For each report, the hospital site, location within the hospital, and time of day (day versus night or weekend) of the event are entered via radio buttons. Possible event locations include the operating room, post-anesthesia care unit (PACU) or preoperative area, labor and delivery, ICU, and remote locations. The provider also submits a free text description of the event, and identifies putative causative mechanism(s) associated with the event via a series of check boxes. The causative mechanisms are based on the Joint Commission patient safety event taxonomy [10]. Since more than one causative mechanism can be selected per event, the total number of causative mechanisms exceeds the total number of events. Given the anonymous nature of the reporting system, and the fact that no patient identifiable information is collected, no post-hoc validation of the causes of the near misses was possible.

### Study design and statistical analysis

Approval for this study was obtained from the UCSF Institutional Review Board, and the requirement for written informed consent was waived.

We retrospectively analyzed near miss reports submitted from January 1, 2009 to December 31, 2011 from the UCSF Medical Center. Near miss reports from other UCSF anesthesia sites were excluded from this analysis since these other sites also have their own internal near miss reporting system such that reports submitted to the departmental near miss system may not be representative of all near misses, and/or the sites do not have intensive care units. Descriptive statistics are stated as frequency and percentage. We characterized the near misses based on the causal mechanisms associated with each report. Using the Joint Commission patient safety event taxonomy, [10] we categorized near misses as technical or non-technical near misses, near misses associated with equipment, near misses associated with systems errors, near misses associated with human errors, and near misses due to a poor culture of safety. Human and systems errors were not mutually exclusive since multiple causative mechanisms could be associated with a single near miss. One author (AKML) identified near misses associated with airway management based on the free text description. We compared causative mechanisms of reports from the ICU with reports from other anesthesia locations. Since the ICU is an alternate location for pre-and post-operative care, we performed a pre-planned subgroup analysis comparing causative mechanisms of ICU near misses with PACU/preoperative near misses. Given the association of ICU near misses and airway management, we performed a post-hoc analysis comparing near misses associated with airway management in the ICU to near misses associated with airway management occurring outside the ICU. Associations were tested using the *Χ*^2^ test, Fisher’s exact test, and logistic regression. Statistical analysis was performed using Stata/IC 12.1 (StataCorp, College Station, TX).

## Results

A total of 15,704 patients were admitted to the adult ICU and 7,672 were admitted to the pediatric ICU during the study period, for a total of 64,307 adult ICU patient days and 62,151 pediatric ICU patient days. There were 63,818 anesthesia cases over the same time period, of which 44,465 (69.7 %) took place in the main operating room, and 811 (1.27 %) occurred in the ICU. Near miss reports totaled 1,811, of which 1,529 (84.4 %) originated in the main operating room and 22 (1.2 %) originated in the ICU. Of the ICU near misses, 18 (82 %) were from the adult ICUs and 4 (18 %) were from the pediatric ICUs. Near miss reports were generated from 2.8 % of non-ICU anesthesia cases, 2.7 % of ICU anesthesia cases, and 0.09 % of ICU admissions. There were 0.17 near misses reported per 1,000 ICU patient days.

Five mechanisms were reported as underlying over half of ICU near misses: failure to execute a task appropriately (16 %), poor communication (12 %), failure to perform a routine task (12 %), poor culture of safety (8 %), and equipment malfunction (8 %) (Table 1). Sixty-four percent of near misses were associated with a systems error, and 41 % were associated with a human error. Compared with near misses from other locations, near misses from the ICU were more likely to occur while on call (45 % vs. 19 %, *p* = 0.001), and were more likely to be associated with airway management (50 % vs. 12 %, *p* < 0.001). ICU near misses were less likely than near misses from other locations to be associated with equipment issues (23 % vs. 48 %, respectively, *p* = 0.02). ICU and non-ICU near misses were equally likely to be associated with human errors (41 % vs. 42 %, respectively, *p* = 0.92), systems errors (64 % vs. 68 %, *p* = 0.7), technical errors (32 % vs. 49 %, *p* = 0.10), and a poor culture of safety (9 % vs. 11 %, *p* = 0.78) (Table 2).

**Table 1:** Causal mechanisms associated with near miss reports originating in the ICU, based on Joint Commission patient safety event taxonomy

	n	%
Skill based: failure to execute a task appropriately	4	16 %
Poor communication	3	12 %
Rule based: failure to perform routine task	3	12 %
Poor culture of safety	2	8 %
Equipment malfunction	2	8 %
Inadequate resources	1	4 %
Time pressure	1	4 %
Faulty design	1	4 %
Faulty construction	1	4 %
Obsolescence	1	4 %
Equipment unavailability	1	4 %
Technical failures beyond control of the institution	1	4 %
Insufficient supervision	1	4 %
Failures related to patient factors beyond control of the institution	1	4 %
Intentional violation	1	4 %
Insufficient training	1	4 %
Total	25	100 %

**Table 2:** Comparison of ICU near misses to near misses from other anesthesia locations

	ICU, n (%)	Other anesthesia locations, n (%)	*p*-value
Time of Day			0.001
Day	12 (55)	1457 (81)	
Call (night/weekend)	10 (45)	332 (19)	
Type of Error			
Human	9 (41)	752 (42)	0.92
Systems	14 (64)	1208 (68)	0.70
Airway-related Error	11 (50)	223 (12)	<0.001
Technical Error	7 (32)	884 (49)	0.10
Equipment Error	5 (23)	855 (48)	0.02
Poor Culture of Safety	2 (9)	196 (11)	0.56

In a pre-planned subgroup analysis, ICU near misses were more likely to occur on call (55 % vs. 45 %, *p* = 0.009) and more likely to be associated with airway management (50 % vs. 13 %, 0.001) than near misses occurring in the preoperative area or PACU. In a post-hoc analysis, airway-related near misses from the ICU did not differ from airway-related near misses occurring outside the ICU in terms of association with human errors, systems errors, technical errors, equipment errors or a poor culture of safety. Airway-related ICU near misses were more likely to have occurred on call than airway-related near misses occurring outside the ICU (64 % vs. 14 %, *p* < 0.001).

Sample free text descriptions of ICU near misses are shown in Table 3. Examples highlight problems in airway management and other anesthesia issues, such as pain management and the care of in-dwelling catheters.

**Table 3:** Sample free text description of near miss events in the ICU

Description of incident	Causal mechanisms
Patient from ICU with [right] radial [arterial] line. [Arterial] line tubing taped tightly around the thumb such that there was a groove in the skin. Patient intubated and unconscious so cannot tell if there is an injury to the digital nerve to thumb [that] may have been compressed for two days.	Poor culture of safety Failure to execute a task appropriately
Patient with difficult mask and intubation extubated evening before major surgery and two teams caring for patient in ICU…did not communicate surgery schedule.	Poor culture of safety
One of our pain service patients had a 3-hour delay between asking for oxycodone for breakthrough pain and when he actually got it…apparently the orders got missed in his transfer between the ICU and the floor.	Time pressure
Hallway blocked on way to ICU - patient with high O2 requirements difficult to ventilate due to gurneys and carts blocking access for second provider to assist. Patient desaturated, [we] stopped and [the patient] recovered.	Faulty design Equipment malfunction
Checking ICU equipment pre-emptively while on call: Glidescope [in first ICU] missing. Glidescope [in another ICU] with reusable handle plugged into end of disposable handle cord . . .so the cord had two handles on either side and no way to plug into the glidescope machine. Glidescope [in yet another ICU] without handles at all.	Equipment unavailability

## Discussion

This study focused on near misses reported by anesthesia providers in the ICU over a three-year period. Although near misses were reported infrequently, our analysis of near miss reports revealed several salient points. First, out of 33 possible causes, five mechanisms explained over half of the near misses reported. Nearly two-thirds of near misses were associated with at least one systems error, and over 40 % of near misses were associated with at least one human error. Errors due to human factors, such as failure of the provider to perform a routine task or complete a task appropriately, deserve special attention since they are associated with increased length of stay, [11] and are difficult to address with conventional patient safety interventions, such as systems modifications and provider education [12]. Other causal mechanisms that were common in this study, such as a poor culture of safety and equipment malfunction, reveal gaps in systems integrity and identify modifications that could improve patient safety, such as organizational changes to promote blame-free reporting and engineering “checks” to identify faulty material goods.

Second, in our study, near misses in the ICU differed from near misses that occurred in other anesthesia locations in several important ways. Near misses in the ICU were more likely to occur at night or on the weekend, as opposed to during the day. The increased incidence of near misses on call may be due to decreased ICU staffing and increased anesthesia involvement in critical care emergencies during those times, and highlights the need to examine the organization, structure and delivery of critical care during off-hours. Additionally, ICU near misses were more likely to be related to airway management than near misses occurring outside of the ICU. This is likely a reflection of the larger proportion of airway-related tasks (such as airway management in the setting of respiratory distress or cardiac arrest) performed by the anesthesia provider in the ICU as compared to other anesthesia locations. However, airway-related events warrant attention since they are frequently associated with physical injury, increased hospital length of stay, and family dissatisfaction, and may be mitigated by adequate ICU staffing and the use of skilled assistants [4]. The use of a recently developed clinical prediction score to identify difficult intubations a priori [13] may further reduce airway-related complications.

Third, ICU near misses were less likely than near misses in other anesthesia locations to be associated with equipment errors. Despite this difference, equipment errors remain an important priority given they may be easily addressed by interventions at the systems-level.

Finally, the rate of near misses reported in the ICU and OR was similar. Although this may be due to a bias in the type of reporting system used, it also suggests anesthesiologist involvement in the ICU may result in lower event rates.

Existing literature on adverse events and near misses in the ICU is primarily based on incident reports submitted by critical care nurses and intensivists [4–9]. To our knowledge, this is the first study evaluating patient safety in the ICU from the unique perspective of the anesthesiologist. The results of our study differ from the results of previous studies, supporting our hypothesis that anesthesia providers can highlight systems weaknesses and causal mechanisms in the ICU not identified by other personnel. As compared to previous work on critical incidents in the ICU by Donchin, *et al.*, a larger proportion of near misses from our study occurred at night or on the weekend, [14] which could imply that decreased staffing in the ICU on off-hours impacts the ability of critical care providers to recognize, react to, and report near misses and adverse events. Additionally, the rate of technical errors in the ICU in our study was lower than previous estimates, [9] which was somewhat surprising given the technical role of the anesthesiologist in airway management and resuscitation. Finally, compared to an analysis of the Intensive Care Unit Safety Reporting System, [4] the proportion of reports related to airway management was much higher in our study. Although this finding is due in part to the larger role in airway management of the anesthesia provider as compared to non-anesthesia critical care providers, it also highlights the gaps in incident reporting that may occur when incident reporting is limited to the ICU team, and suggests that specialist consultants should be engaged in the ICU patient safety movement.

Our study has several limitations. First, we are limited by our small sample size. The number of events reported per 1,000 ICU patient days is markedly lower than that reported in previous studies [5–9, 15, 16]. However, anesthesia providers function in a different role in the ICU than critical care providers; for instance, anesthesia providers may only be involved in the care of ICU patients during procedures, transport, or emergency situations. Thus, it may not be reasonable to expect similar event rates. Of note, the number of near miss reports per anesthesia case was similar in the ICU and outside the ICU, suggesting similar reporting patterns. Nonetheless, since our reporting system is voluntary, we are unlikely to be capturing all near misses. Additionally, our reporting system, in which providers submit reports via an online interface, may not be the ideal mechanism for capturing critical incidents. Mandatory online reporting, [17] paper collection cards, [5, 7–9, 18], real-time audits, [19] facilitated incident monitoring, [20] and direct observation [16] may increase the number of events captured.

Second, our near miss system captures limited information in order to minimize the time required to report an event. As such, additional data that could enhance our analysis, such as the provider’s role in the care of the patient, or the patient’s age and comorbidities are not available.

Additionally, the differences observed between ICU near misses and near misses from other anesthesia locations may simply be due to differences in the nature of care in the ICU as compared to other locations where anesthesia is provided. And, differences between the causal mechanisms associated with our near misses and those previously published in the literature may be confounded by heterogeneity in the definitions of the mechanisms being studied. Furthermore, since our near miss system collects reports solely from providers within the department of anesthesia, it is not possible to compare reports submitted by anesthesia providers to those submitted by other specialists.

Finally, since our data were collected from a single academic anesthesia department with a large number of critical care trained faculty, our findings may not be generalizable to other institutions.

## Conclusions

To our knowledge, this is the first analysis of near miss reports associated with ICU care from the perspective of the anesthesia provider. A few causal mechanisms explained the majority of ICU near misses, providing targets for quality improvement. Errors associated with airway management may be more common in the ICU than other anesthesia locations; further efforts to understand the dangers of airway management in the ICU are needed. Specialist consultants may be able to identify systems weaknesses not identified by critical care providers, and should be engaged in the ICU patient safety movement.
